# Metformin prevents mandibular bone loss in a mouse model of accelerated aging by correcting dysregulated AMPK-mTOR signaling and osteoclast differentiation

**DOI:** 10.1016/j.jot.2024.03.001

**Published:** 2024-05-28

**Authors:** Boyang Liu, Jiao Zhang, Jinge Zhang, Xiaolei Ji, Rong Wang, Aixiu Gong, Dengshun Miao

**Affiliations:** a Department of Human Anatomy, Histology and Embryology, The Research Center for Bone and Stem Cells, Nanjing Medical University, Nanjing, PR China; b Department of Plastic Surgery, Affiliated Friendship Plastic Surgery Hospital of Nanjing Medical University, Nanjing Medical University, Nanjing, PR China; c Department of Stomatology, The Affiliated BenQ Hospital of Nanjing Medical University, Nanjing, PR China; d Department of Stomatology, Children's Hospital of Nanjing Medical University, Nanjing, PR China

**Keywords:** AMPK-mTOR, Bmi1 deficiency, Mandibular bone loss, Metformin, p53- Stfa1

## Abstract

**Background:**

Age-related mandibular osteoporosis frequently causes loose teeth, difficulty eating, and disfiguration in elders. Bmi1^−/−^ mice displaying accelerated skeletal aging represent a useful model for testing interventions against premature jaw bone loss. As an anti-aging agent, metformin may ameliorate molecular dysfunction driving osteoporosis pathogenesis. We explored the mechanisms of mandibular osteopenia in Bmi1^−/−^ mice and prevention by metformin treatment.

**Methods:**

Three mouse groups were utilized: wild-type controls, untreated Bmi1^−/−^, and Bmi1^−/−^ receiving 1 g/kg metformin diet. Mandibular bone phenotype was assessed by X-ray, micro-CT, histology, and immunohistochemistry. AMPK-mTOR pathway analysis, senescence markers, osteoblast and osteoclast gene expression were evaluated in jaw tissue. Osteoclast differentiation capacity and associated signaling molecules were examined in cultured Bmi1^−/−^ bone marrow mononuclear cells ± metformin.

**Results:**

Bmi1 loss reduced mandible bone density concomitant with decreased AMPK activity, increased mTOR signaling and cellular senescence in jaw tissue versus wild-type controls. This was accompanied by impaired osteoblast function and upregulated osteoclastogenesis markers. Metformin administration normalized AMPK-mTOR balance, oxidative stress and senescence signaling to significantly improve mandibular bone architecture in Bmi1^−/−^ mice. In culture, metformin attenuated excessive osteoclast differentiation from Bmi1^−/−^ marrow precursors by correcting dysregulated AMPK-mTOR-p53 pathway activity and suppressing novel pro-osteoclastogenic factor Stfa1.

**Conclusions:**

Our study newly demonstrates metformin prevents accelerated jaw bone loss in a premature aging murine model by rectifying molecular dysfunction in cellular energy sensors, redox state, senescence and osteoclastogenesis pathways. Targeting such age-associated mechanisms contributing to osteoporosis pathogenesis may help maintain oral health and aesthetics in the growing elderly population.

**Translational potential:**

The pronounced mandibular osteopenia exhibited in Bmi1^−/−^ mice represents an accelerated model of jaw bone deterioration observed during human aging. Our finding that metformin preserves mandibular bone integrity in this progeroid model has important clinical implications. As an inexpensive oral medication already widely used to manage diabetes, metformin holds translational promise for mitigating age-related osteoporosis. The mandible is essential for chewing, swallowing, speech and facial structure, but progressively loses bone mass and strength with advancing age, significantly impacting seniors' nutrition, physical function and self-image. Our results suggest metformin's ability to rectify cellular energy imbalance, oxidative stress and osteoclast overactivity may help maintain jaw bone health into old age. Further research is still needed given metformin's multifaceted biology and bone regulation by diverse pathways. However, this preclinical study provides a strong rationale for clinical trials specifically examining mandibular outcomes in elderly subjects receiving standard metformin treatment for diabetes or prediabetes. Determining if metformin supplementation can prevent or delay oral disability and disfigurement from senescent jaw bone loss in the growing aged population represents an important public health priority. In summary, our mechanistic findings in a genetic mouse model indicate metformin merits investigation in rigorous human studies for alleviating morbidity associated with age-related mandibular osteoporosis.

## Graphical abstract

**Figure:**
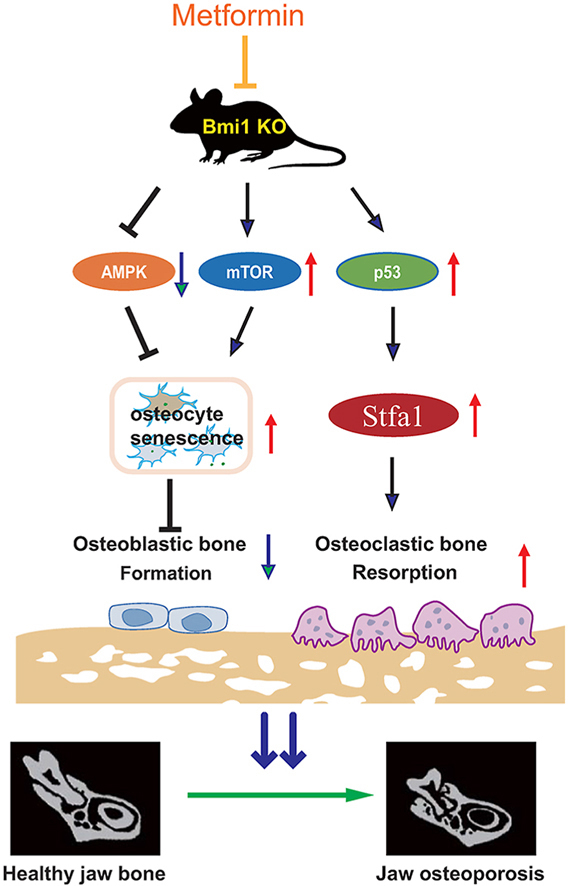

## 1 Introduction

Age-related bone loss and osteoporosis are major public health issues, significantly impacting quality of life in the elderly population [1]. The jaw bones, including the mandible, undergo progressive bone loss with aging similar to other skeletal sites [2]. This can lead to loose teeth, difficulty eating, and altered facial appearance in seniors. Studies of genetic mouse models indicate that molecular pathways regulating cellular senescence and longevity also modulate the pace of age-related bone loss [3]. Hence, targeting these regulatory nodes may offer therapeutic strategies to maintain jaw bone health into old age.

Bmi1 is an epigenetic regulator that represses expression of the Ink4a/Arf locus encoding cell cycle inhibitors p16^Ink4a^ and p19^Arf^ [4]. Through this activity, Bmi1 stimulates self-renewal of adult stem cells and inhibits premature aging [5]. Consistent with this function, mice deficient in Bmi1 (Bmi1^−/−^) display multiple symptoms of premature aging including significant bone loss and osteoporosis throughout the skeleton as early as 1 month old [6,7]. Our group has further shown Bmi1^−/−^ mice exhibit pronounced osteopenia in the mandible by 5 weeks old, with dramatic loss of alveolar bone and thinner cortical bone [8]. At the cellular level, Bmi1^−/−^ jaw bones have impaired bone formation by osteoblasts coupled with excessive osteoclastic bone resorption. Hence, Bmi1^−/−^ mice represent an accelerated model of age-related mandibular bone loss useful for testing preventative interventions.

Metformin (MET) is an oral biguanide drug used worldwide for treating type 2 diabetes. Recently, metformin has gained significant interest for its potential to promote healthy longevity and ameliorate age-related degeneration across taxa [9,10]. At the molecular level, metformin indirectly activates AMP-activated protein kinase (AMPK), a central sensor of cellular energy status that stimulates catabolism and inhibits anabolic mTORC1 signaling [11]. Through this mechanism, metformin is believed to rectify energy imbalance associated with aging and reprogram cellular metabolism. Intriguingly, several studies indicate metformin can counter bone loss in aged mice, although its bone effects likely involve additional pathways beyond AMPK-mTOR signaling [[12], [13], [14]]. Nevertheless, we hypothesized metformin may preserve mandibular bone integrity in the context of accelerated aging by targeting dysregulation of AMPK-mTOR and osteoblast-osteoclast balance.

In this study, we tested if metformin could prevent mandibular bone loss in Bmi1^−/−^ mice as a model of age-related osteoporosis. Using histology, immunohistochemistry, RNA sequencing, cell culture, and luciferase assays, we investigated the mechanism of mandibular bone loss in Bmi1^−/−^ mice and rescue by metformin treatment. Our results indicate loss of Bmi1 reduces AMPK activity and increases mTOR signaling in jaw bone to stimulate osteoclast differentiation and bone resorption through p53 and Stfa1. Metformin preserved mandibular bone density and architecture in Bmi1^−/−^ mice by correcting AMPK-mTOR imbalance to inhibit excessive osteoclastic activity. Our results provide insight into cell senescence pathways driving jaw osteoporosis pathogenesis. Pharmacological agents like metformin capable of alleviating molecular dysfunction associated with senescence may represent promising therapeutic strategies. Overall, our findings shed light on the mechanism of bone protective effects of metformin through targeting fundamental aging processes.

## 2 Materials and methods

### 2.1 Animal models

All mice were housed in accredited experimental animal facilities at Nanjing Medical University. The study protocol received approval from the University Ethics Committee. Bmi1^−/−^ mice, utilized in the research, were generated as described in previous studies [6].

### 2.2 Animal treatment and dietary MET supplementation

Purified metformin (Sigma) was used as a medicated feed additive, and the MET-supplemented diet was produced by Jiangsu Synergy Biotechnology Co., Ltd., China. Mice were categorized into three groups, each comprising six mice, and treated as follows:

Normal diet (WT) group: Wild-type littermates were weaned at 3 weeks and fed a regular diet for 3 weeks.

Normal diet (Bmi1^−/−^) group: Bmi1 gene knockout littermates were weaned at 3 weeks and fed a regular diet for 3 weeks.

MET-supplemented diet (Bmi1^−/−^ + MET) group: Bmi1 gene knockout littermates were weaned at 3 weeks and fed a diet supplemented with MET [15] (1 g/kg MET added to a regular diet) for 3 weeks. After 3 weeks, six mice from each group were sacrificed for further analysis.

### 2.3 Imaging analysis

Mandibles were carefully dissected from all soft tissues as detailed in Ref. [16]. Subsequently, specimens underwent X-ray photography and micro-CT analysis [17].

### 2.4 Histology

Tissues were collected and processed as previously outlined [18,19]. Briefly, paraffin-embedded blocks were sectioned and stained with hematoxylin & eosin (H&E) and histochemical staining for total collagen or tartrate-resistant acid phosphatase (TRAP) expression.

### 2.5 Immunohistochemical staining

Sections from paraffin-embedded samples were stained for the proteins p16, β-Gal and SOD2 following a protocol outlined in earlier studies [20]. Briefly, sections were deparaffinized, rehydrated, blocked with hydrogen peroxide (6%), and incubated overnight at 4 °C with primary antibodies (β-Galactosidase, SOD2 from Abcam, p16 from Cell Signaling Technology). After primary antibody staining, slides were washed, incubated with secondary antibodies (Sigma biotinylated goat anti-rabbit IgG and goat anti-mouse IgG), and subjected to further processing as described.

### 2.6 Western Blot analysis

Total protein from mouse mandibles was collected, and equal amounts (20 μg) were loaded for SDS-PAGE separation. Membranes were probed with various primary antibodies, and chemiluminescence detection was performed as previously described [21].

### 2.7 Real-time quantitative PCR analysis

Total RNA extraction and cDNA synthesis were carried out using TRIzol reagent (Invitrogen) and the PrimeScriptTM 1st Strand cDNA Synthesis Kit (Takara Bio), respectively. Gene expression was analyzed using real-time PCR with specific primers (Table 1).

**Table 1:** Primers used for quantitative real-time PCR.

Name	Species	Forward	Reverse
Gapdh	Mouse	AGGTCGGTGTGAACGGATTTG	TGTAGACCATGTAGTTGAGGTCA
Rankl	Mouse	CAGCATCGCTCTGTTCCTGTA	CTGCGTTTTCATGGAGTCTCA
Opg	Mouse	ACCCAGAAACTGGTCATCAGC	CTGCAATACACACACTCATCACT
p16	Mouse	CGCAGGTTCTTGGTCACTGT	TGTTCACGAAAGCCAGAGCG
p21	Mouse	CCTGGTGATGTCCGACCTG	CCATGAGCGCATCGCAATC
p53	Mouse	GAAGTCCTTTGCCCTGAAC	CTAGCAGTTTGGGCTTTCC

**Table 2:** Primers used for CUT&RUN.

Name	Forward	Reverse
Stfa1 site	GTTATTCGTGCCCTTGCTCA	GAATGGGGAAATGAGTGTGGC

**Table 3:** Indicated promoter sequences of Stfa1 cloned to pGL4.10

Stfa1 promoter- pGL4.10
GTATTTTCTTTAAAGATCTCTTTAAGCGTTAACTTATTTAGAAACACAGGTTATTCGTGCCCTTGCTCATATGACACAATATGATGGCATTATTGGCACAGAGACTGCAGGGAGGAGCCATGCTTACCACACCTGAGTCTGGGCACACATGCTTCCTCATGTCCCCCACCACATTTACCCCCAACTCCACTCAGCATTTCCTGTAATGGATGCAGTAAGACTTGCCTAAGGCCACACTCATTTCCCCATTCCCTCAGATAATGTGGAGATAAGACACTAGAGGAGGAGCTTTCGGATACCTTTAAAACCCTGAGTCTCAGTGCTTTGCAGG
**Stfa1 promoter mutant- pGL4.10**
GTATTTTCTTTAAAGATCTCTTTAAGCGTTAACTTATTTAGAAACACAGGTTATTCGTGCCCTTGCTCATATGACACAATATGATGGCATTATTGGCACAGAGACTGCAGGGAGGAGCCATGCTTACCACACCTGAGTCTGGGCAAAAAAAAAAAAAAAAAAACCCCACCACATTTACCCCCAACTCCACTCAGCATTTCCTGTAATGGATGCAGTAAGACTTGCCTAAGGCCACACTCATTTCCCCATTCCCTCAGATAATGTGGAGATAAGACACTAGAGGAGGAGCTTTCGGATACCTTTAAAACCCTGAGTCTCAGTGCTTTGCAGG

### 2.8 Cell cultures

Mouse bone marrow-derived mesenchymal stem cells (BM-MSCs) were isolated from the femur and tibia and subsequently cultured in α-MEM medium. The medium was supplemented with 10% fetal bovine serum (FBS) and 1% penicillin/streptomycin. Mouse bone marrow mononuclear cells (BMMs) were isolated from the femur and tibia and cultured in Dulbecco's Modified Eagle's Medium (DMEM) supplemented with 10% fetal bovine serum (FBS), 1% penicillin/streptomycin, and 50 ng/mL macrophage colony-stimulating factor (M-CSF) from PeproTech. The adherent cells were treated with 2.5 mM or 5 mM MET (Sigma), 500 ng/mL rapamycin (Mce), 5 mM 3-MA (Mce), and 20 μ M hydrogen peroxide (H_2_0_2_) or 20 μ M pifithrin-alpha (PFT- α) from Mce. Subsequent investigations were conducted to assess the effects of these treatments.

### 2.9 Osteoclast formation assay

Osteoclast differentiation from mouse BMMs was induced, and TRAP staining was performed as previously described [22].

### 2.10 siRNA, lentivirus construction, and transfection

Mouse BMMs were transfected with Stfa1 siRNA or non-targeting siRNA for 24 h. For Stfa1 overexpression, lentiviral particles were generated and used for cell infection.

### 2.11 CUT&RUN qPCR

CUT&RUN-qPCR experiments were conducted using the CUT&RUN kit (vazyme) with rabbit anti-p53 antibodies. Enriched DNA was used for qPCR detection of predicted STFA1 binding (Table 2).

### 2.12 Dual luciferase assay

STFA1 promoters of indicated length and mutants were synthesized and cloned into pGL3-basic (Promega) (Table 3). HEK293T cells were co-transfected with p53-driven luciferase, pcDNA3.1-STFA1, and pRL-TK, and luciferase activities were measured.

### 2.13 Statistical analysis

Data are presented as mean ± SD. Statistical analyses were performed using GraphPad Prism (version 8.0). Two-tailed Student's t-test was used for group comparisons, and one-way or two-way ANOVA was used for multiple comparisons. P values < 0.05, <0.01, and <0.001 were considered statistically significant (*, **, ***).

## 3 Results

### 3.1 Bmi1 deficiency leads to dysregulation of AMPK-mTOR activity in mandibular tissue, BM-MSCs and BMMs

**Figure 1:**
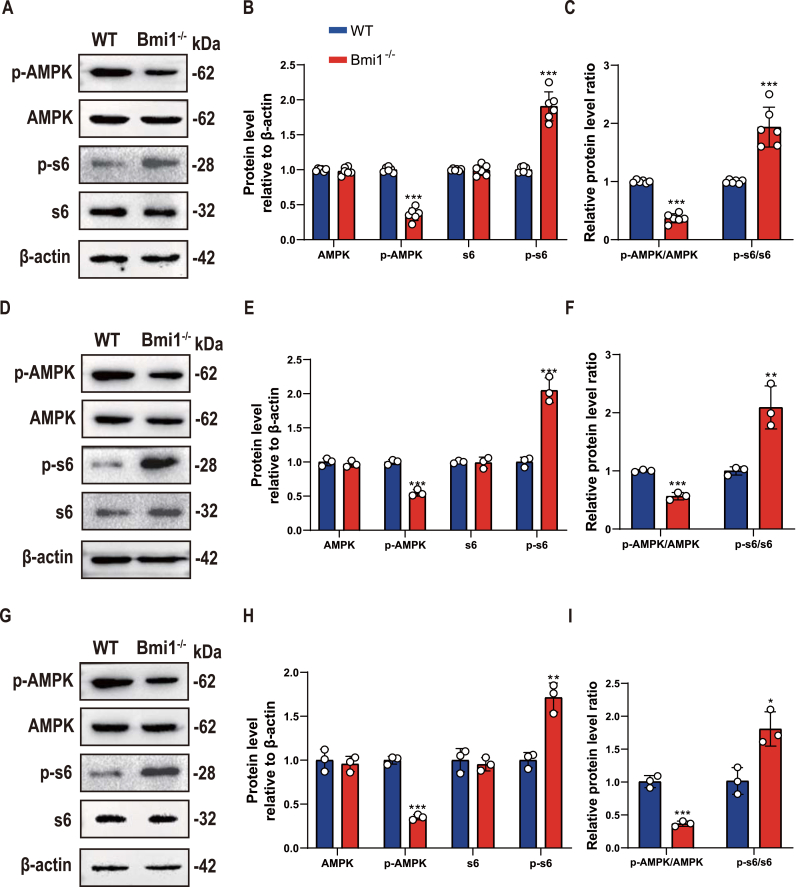
Bmi1 deficiency leads to dysregulation of AMPK-mTOR activity in mandibular tissue, BM-MSCs, and BMMs (A) Western blots of mandibular extracts were performed to assess the expression of AMPK, p-AMPK, s6, and p-s6. (B) Densitometric analysis of protein levels relative to β-actin, expressed as a percentage of WT mice levels. (C) Ratio of protein levels relative to β-actin of p-AMPK/AMPK, p-s6/s6 in the two groups mentioned above. (D) Western blots for AMPK, p-AMPK, s6, and p-s6 protein expression levels in BMSCs extracted from WT and Bmi1−/− mice. (E) Densitometric analysis of protein levels relative to β-actin, expressed as a percentage of WT mice levels. (F) Ratio of protein levels relative to β-actin of p-AMPK/AMPK, p-s6/s6 in the two groups mentioned above. (G) Western blots for AMPK, p-AMPK, s6, and p-s6 protein expression levels in BMMs extracted from WT and Bmi1^−/−^ mice. (H) Densitometric analysis of protein levels relative to β-actin, expressed as a percentage of WT mice levels. (I) Ratio of protein levels relative to β-actin of p-AMPK/AMPK, p-s6/s6 in the two groups mentioned above. Values are mean ± S.E.M. of 3 determinations per group. *: P < 0.05, **: P < 0.01, ***: P < 0.001 compared with WT mice; ###: P < 0.001 compared with Bmi1^−/−^ mice.

To determine whether Bmi1 deficiency leads to dysregulation of AMPK-mTOR activity in mandibular tissue, bone marrow mesenchymal stem cells (BM-MSCs) and bone marrow mononuclear cells (BMMs), we used Western blot to detect changes in protein expression levels of AMPK, p-AMPK, s6 (reflecting mTOR expression level), and p-s6 in wild-type and Bmi1^−/−^ mouse mandibular tissue, BM-MSCs and BMMs. The results showed that compared with wild-type mice, there was no significant change in AMPK and s6 protein expression levels in mandibular tissue, BM-MSCs and BMMs of Bmi1^−/−^ mice, while p-AMPK protein expression levels and p-AMPK/AMPK ratio were significantly decreased and p-s6 protein expression levels and p-s6/s6 ratio were significantly increased in mandibular tissue, BM-MSCs and BMMs of Bmi1^−/−^ mice (Fig. 1A–I). These results suggest that Bmi1 deficiency can inhibit AMPK activity and increase mTOR activity in mandibular tissue, BM-MSCs and BMMs.

### 3.2 Metformin significantly improves mandibular osteoporosis caused by Bmi1 deficiency

**Figure 2:**
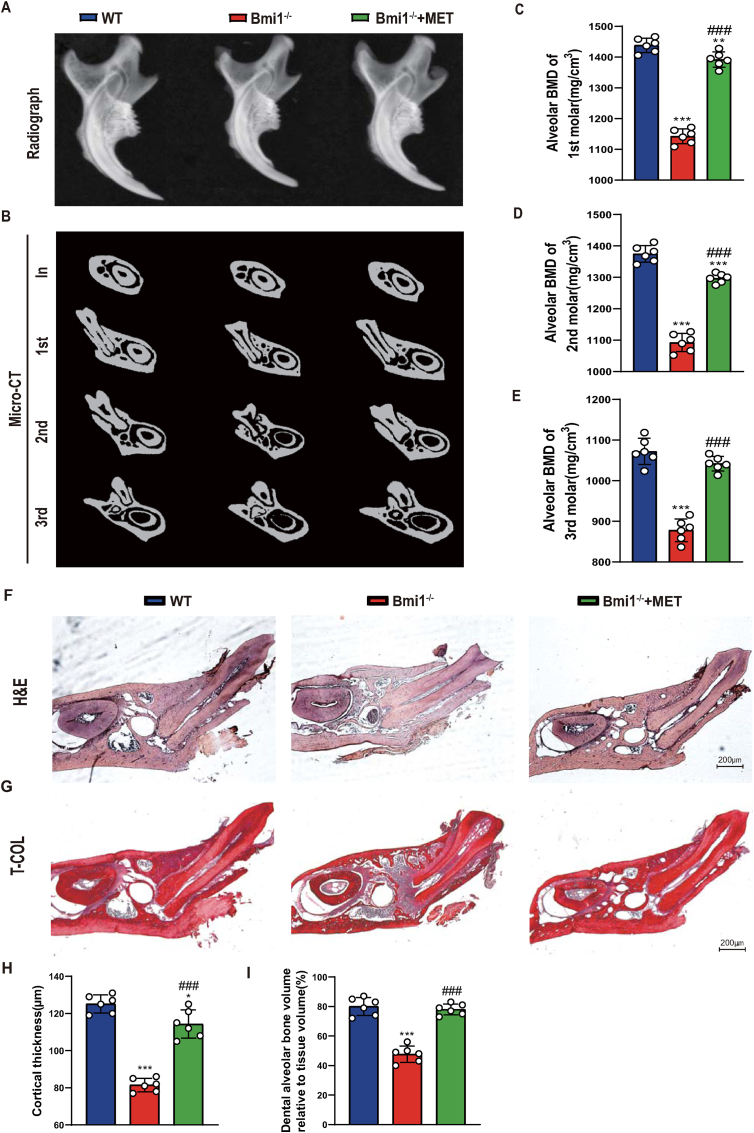
Metformin significantly improves mandibular osteoporosis caused by Bmi1 deficiency (A) Representative radiographs of mandibles from 6-week-old WT, Bmi1^−/−^, Bmi1^−/−^ + MET mice. (B) Representative images of micro-CT-scanned sections through the incisors in front of the first molar (In), the first (1st), second (2nd), and third (3rd) molars. (C–E) Alveolar BMD of 1st, 2nd, and 3rd molars. (F) Representative micrographs of decalcified paraffin-embedded sections through the first molars and the incisors from the three groups were stained histochemically for hematoxylin and eosin (H&E), (G) total collagen (T-Col) (50x) (H) Cortical thickness, (I) Dental alveolar bone volume relative to tissue volume (BV/TV, %). Values are mean ± S.E.M. of 6 determinations per group. *: P < 0.05, **: P < 0.01, ***: P < 0.001 compared with WT mice; ###: P < 0.001 compared with Bmi1^−/−^ mice.

Previous studies have suggested that metformin exerts life-extending and health-promoting effects of aging by activating key longevity pathways such as AMPK and inhibiting pro-aging pathways such as mTOR. Therefore, in this study, we proposed to study whether metformin could rescue mandibular osteoporosis caused by Bmi1 deficiency. We fed Bmi1^−/−^ mice with metformin diet, and used X-ray photography, Micro-CT scanning and histological methods to compare and analyze the differences in alveolar bone density, bone mass and cortical thickness of the mandible between them and normal dietary wild-type or Bmi1^−/−^ mice. The results showed that the alveolar bone density, cortical thickness and alveolar bone volume in the mandible of Bmi1^−/−^ mice were significantly lower than those of wild-type mice, while those in the mandible of metformin-supplemented Bmi1^−/−^ mice were significantly higher than those of the normal dietary Bmi1^−/−^ mice, but had not yet reached wild-type levels (Fig. 2A–I). These results indicate that metformin can significantly rescue mandibular osteoporosis caused by Bmi1 deficiency.

### 3.3 Metformin significantly improves decreased osteoblast bone formation and increased osteoclast bone resorption caused by Bmi1 deficiency

**Figure 3:**
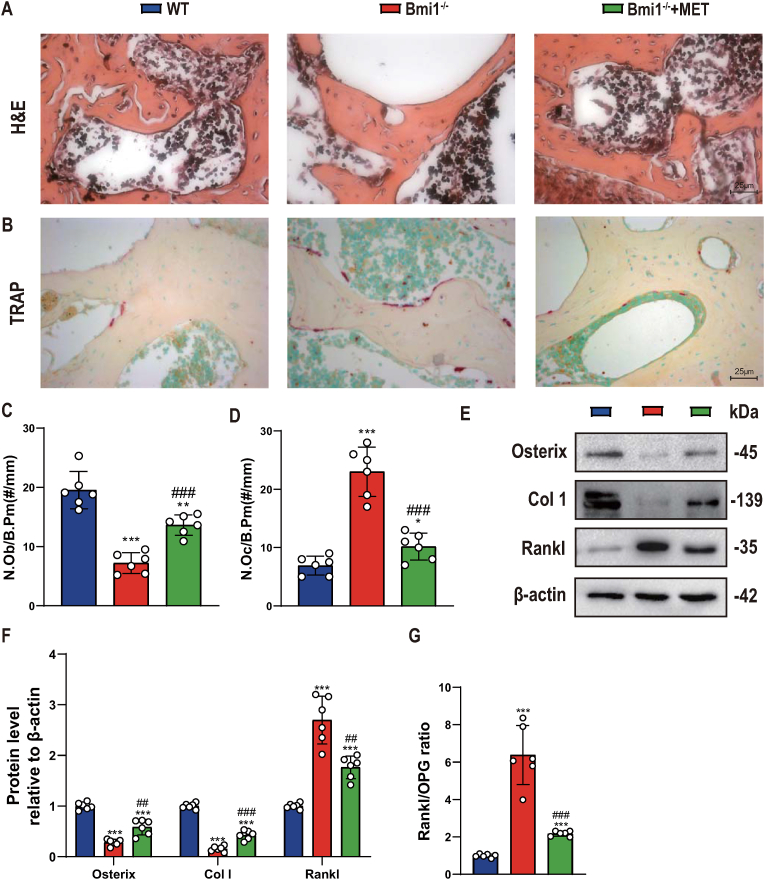
Metformin significantly improves decreased osteoblast bone formation and increased osteoclast bone resorption caused by Bmi1 deficiency Representative micrographs of decalcified paraffin-embedded sections through the first molars and the incisors from the three groups were stained histochemically for (A) Micrographs of H&E staining of alveolar bone. (B) Histochemically for tartrate-resistant acid phosphatase (TRAP). (C) Number of positive osteoblasts of alveolar bone. (D) Number of TRAP-positive osteoclasts of alveolar bone. (E) Western blots of mandibular extracts were performed for expression of osterix, Col 1, Rankl. (F) Densitometric analysis of protein levels relative to β-actin, expressed as a percentage of WT mice levels. (G) qRT-PCR of tissue extracts of mandibles for expression of Rankl and OPG. Messenger RNA expression assessed by real-time qRT-PCR is calculated as a ratio relative to Gapdh, and expressed relative to WT mice. (H) Rankl/OPG ratio of mRNA levels. Values are mean ± S.E.M. of 6 determinations per group. *: P < 0.05, **: P < 0.01, ***: P < 0.001 compared with WT mice; ##: P < 0.01, ###: P < 0.001 compared with Bmi1^−/−^ mice.

In order to clarify whether the rescue effect of metformin on mandibular osteoporosis caused by Bmi1 deficiency is related to changes in alveolar bone turnover, we used histopathological, Western blot and qRT-PCR methods to compare and analyze changes in osteogenic, osteoclast-related indicators in mandibular tissue of the above 3 groups of mice. The results showed that the number of osteoblasts, mRNA expression levels of osterix, type I collagen and osteoprotegerin (OPG) in mandibular tissue of Bmi1^−/−^ mice were significantly lower than those of wild-type mice, while those in mandibular tissue of metformin-supplemented Bmi1^−/−^ mice were significantly higher than those of the normal dietary Bmi1^−/−^ mice, but had not yet reached wild-type levels (Fig. 3A–G). In contrast, TRAP positive osteoclasts, Rankl protein and mRNA expression levels and Rankl/OPG ratio in mandibular tissue of Bmi1^−/−^ mice were significantly higher than those of wild-type mice, while those in mandibular tissue of metformin-supplemented Bmi1^−/−^ mice were significantly lower than those of the normal dietary Bmi1^−/−^ mice, but had not yet reached wild-type levels (Fig. 3A–G). These results indicate that metformin can stimulate osteoblast bone formation and inhibit osteoclast bone resorption to correct mandibular osteoporosis caused by Bmi1 deficiency.

### 3.4 Metformin significantly inhibits decreased antioxidant capacity and increased osteocyte senescence caused by Bmi1 deficiency

**Figure 4:**
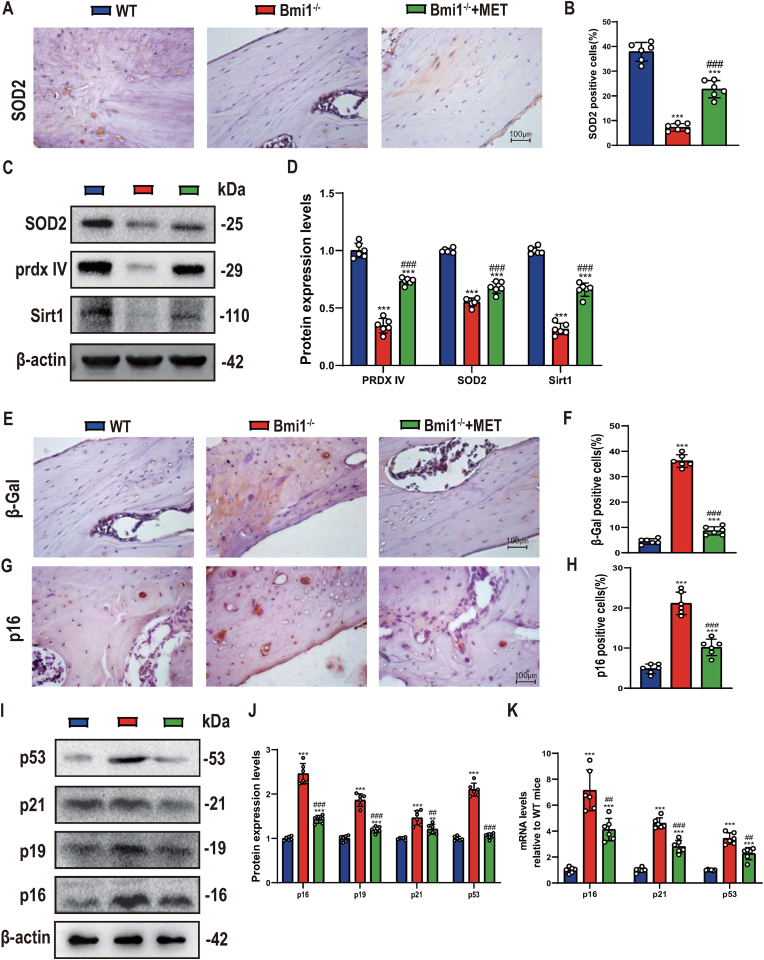
Metformin significantly inhibits decreased antioxidant capacity and increased osteocyte senescence caused by Bmi1 deficiency Representative micrographs of decalcified paraffin-embedded sections through the first molars and the incisors from 6-week-old WT, Bmi1^−/−^, Bmi1^−/−^ + MET mice stained immunohistochemically for (A) SOD2(400x). (B) SOD2 positive cells%. (C) Western blots of mandibular extracts were performed for expression of SOD2, prdx IV, Sirt1. (D) Densitometric analysis of protein levels relative to β-actin, expressed as a percentage of WT mice levels. (E) Immunohistochemical staining for β-Gal, p16, p53. (F) β-Gal positive cells%. (G) p16 positive cells%. (H) p53 positive cells%. (I) Western blots of mandibular extracts were performed for expression of p16, p19, p21, and p53. (J) Densitometric analysis of protein levels relative to β-actin, expressed as a percentage of WT mice levels. (K) qRT-PCR of tissue extracts of mandibles for expression of p16, p21, and p53. Messenger RNA expression assessed by real-time qRT-PCR is calculated as a ratio relative to Gapdh, and expressed relative to WT mice. Values are mean ± S.E.M. of 6 determinations per group. **: P < 0.01, ***: P < 0.001 compared with WT mice; ##: P < 0.01, ###: P < 0.001 compared with Bmi1^−/−^ mice.

In order to clarify whether the significant correction of decreased osteoblast bone formation caused by metformin is related to changes in antioxidant capacity and osteocyte senescence in mandibular tissue, we used immunohistochemistry, Western blot and qRT-PCR methods to compare and analyze changes in oxidative stress and cellular senescence related indicators in mandibular tissue of the above 3 groups of mice. The results showed that the percentage of superoxide dismutase 2 (SOD2) positive cells, protein expression levels of SOD2, peroxiredoxin IV (Prdx IV) and Sirt1 were significantly lower in mandibular tissue of Bmi1^−/−^ mice than in wild-type mice, while those in mandibular tissue of metformin-supplemented Bmi1^−/−^ mice were significantly higher than those of the normal dietary Bmi1^−/−^ mice, but had not yet reached wild-type levels (Fig. 4A–D). Cell senescence-related indicators including β-Gal and p16 positive osteoblast percentages, protein expression of p16, p19, p21, and p53 as well as mRNA expression levels of p16, p21 and p53 were significantly increased in mandibular tissue of Bmi1^−/−^ mice compared to wild-type mice, while those in mandibular tissue of metformin-supplemented Bmi1^−/−^ mice were significantly lower than those of the normal dietary Bmi1^−/−^ mice, but had not yet reached wild-type levels (Fig. 4E–K). These results indicate that metformin can upregulate antioxidant enzyme expression and inhibit oxidative stress and osteocyte senescence in mandibular tissue.

### 3.5 Metformin corrects dysregulation of AMPK-mTOR-p53 signaling and increased osteoclast differentiation in Bmi1 deficient BMMs

**Figure 5:**
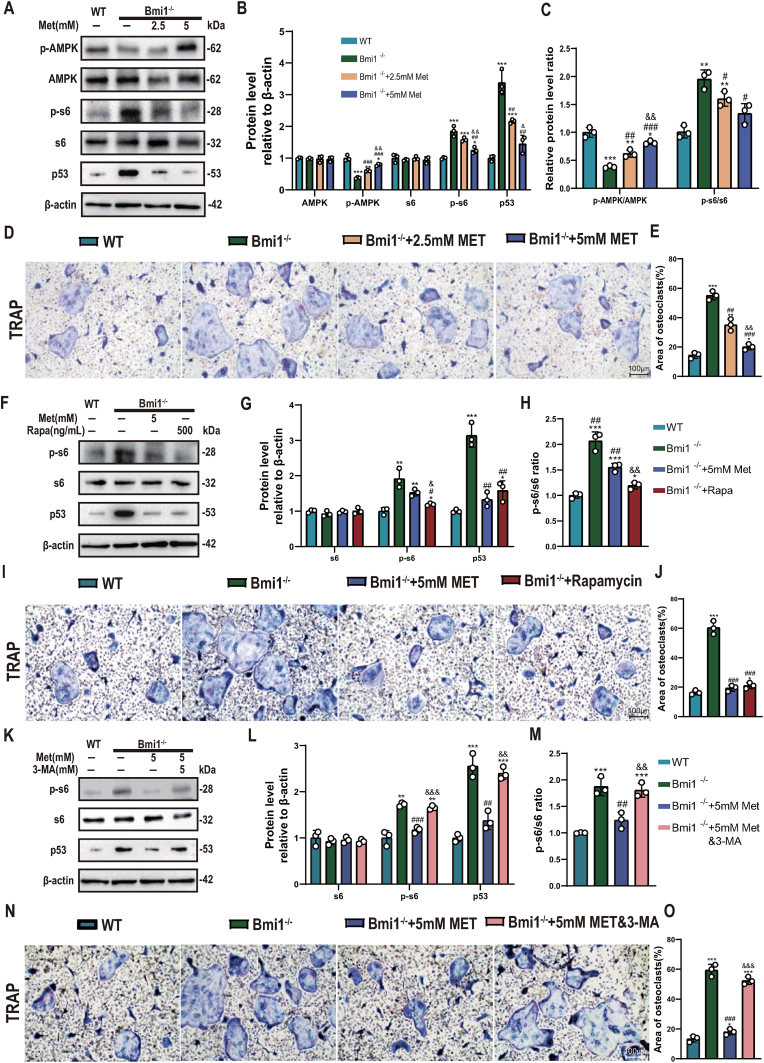
Metformin corrects dysregulation of AMPK-mTOR-p53 signaling and increased osteoclast differentiation in Bmi1 deficient BMMs (A) Western blots for AMPK, p-AMPK, s6, p-s6, and p53 protein expression levels of BMMs extracted from WT and Bmi1^−/−^ mice and co-cultured with 2.5 mM or 5 mM MET. (B) Densitometric analysis of protein levels relative to β-actin, expressed as a percentage of the levels of BMMs of WT mice. (C) Ratio of protein levels relative to β-actin of p-AMPK/AMPK, p-s6/s6 of 4 groups above. (D) TRAP staining after osteoclast induction of 4 groups above. (E) Area of osteoclasts of 4 groups above. (F) Western blots for s6, p-s6, and p53 protein expression levels of BMMs extracted from WT and Bmi1^−/−^ mice and co-cultured with 5 mM MET or 500 ng/mL rapamycin. (G) Densitometric analysis of protein levels relative to β-actin, expressed as a percentage of the levels of BMMs of WT mice. (H) Ratio of protein levels relative to β-actin of p-s6/s6 of 4 groups above. (I) TRAP staining after osteoclast induction of 4 groups above(100x). (J) Area of osteoclasts of 4 groups above. (K) Western blots for s6 and p-s6 protein expression levels of BMMs extracted from WT and Bmi1^−/−^ mice and co-cultured with 5 mM MET or 5 mM MET+5 mM 3-MA. (L) Densitometric analysis of protein levels relative to β-actin, expressed as a percentage of the levels of BMMs of WT mice. (M) Ratio of protein levels relative to β-actin of p-s6/s6 of 4 groups above. (N) TRAP staining after osteoclast induction of 4 groups above(100x). (O) Area of osteoclast of 4 groups above. Values are mean ± S.E.M. of 3 determinations per group. *: P < 0.05, **: P < 0.01, ***: P < 0.001 compared with WT; #: P < 0.05, ##: P < 0.01, ###: P < 0.001 compared with Bmi1^−/−^; &: P < 0.05, &&: P < 0.01, &&&: P < 0.001 compared with Bmi1^−/−^+2.5 mM MET or Bmi1^−/−^+5 mM MET.

In order to clarify whether the improvement of increased osteoclast bone resorption caused by metformin is related to the correction of dysregulation of AMPK-mTOR-p53 signaling and increased osteoclast differentiation in BMMs caused by Bmi1 deficiency, we isolated BMMs from bilateral femurs and tibias of 6-week-old wild-type and Bmi1^−/−^ mice, cultured them under conditions with or without 2.5 mM or 5 mM metformin, 500 ng/mL rapamycin, or 5 mM metformin plus 5 mM mTOR activator 3-MA, and used Western blot to detect changes in protein expression levels of AMPK, p-AMPK, s6, p-s6 and p53. BMMs were also cultured under osteoclast induction conditions and TRAP cytochemical staining was used to analyze changes in osteoclast differentiation. The results showed that metformin corrected Bmi1 deficiency-induced downregulation of p-AMPK and p-AMPK/AMPK ratio, upregulation of p-s6, p-s6/s6 ratio and p53, and increased osteoclast differentiation of BMMs in a dose-dependent manner (Fig. 5A–E). Rapamycin, similar to metformin, also corrected upregulation of p-s6, p-s6/s6 ratio and p53 and increased osteoclast differentiation caused by Bmi1 deficiency (Fig. 5F–J), while the mTOR activator 3-MA could block the corrective effect of metformin on upregulation of p-s6, p-s6/s6 ratio and p53 and increased osteoclast differentiation caused by Bmi1 deficiency (Fig. 5K-O). These results indicate that Bmi1 deficiency stimulates osteoclast differentiation of BMMs by inactivating AMPK, activating mTOR signaling and upregulating p53, while metformin can correct increased osteoclast differentiation in Bmi1 deficient BMMs by activating AMPK, inactivating mTOR signaling and downregulating p53.

### 3.6 Metformin corrects increased osteoclast differentiation in Bmi1 deficient BMMs by downregulating Stfa1

**Figure 6:**
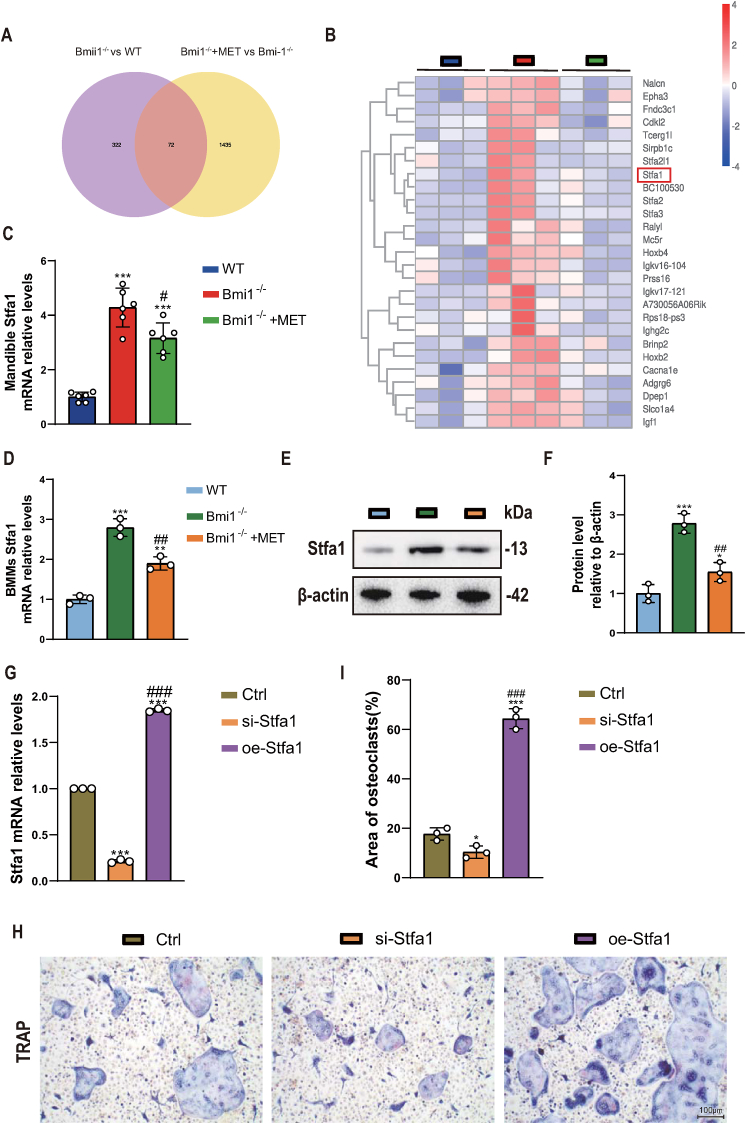
Metformin Corrects Increased Osteoclast Differentiation in Bmi1-Deficient BMMs by Downregulating Stfa1 (A) Venn diagram illustrating RNA-sequencing results in mandibular tissue from six-week-old WT, Bmi1^−/−^, and Bmi1^−/−^ + MET mice. (B) Heatmap of RNA-sequencing data displaying differential gene expression in mandibular tissue from the aforementioned three groups. (C) qRT-PCR validation of Stfa1 mRNA expression levels in the mandibular tissue of the three groups. (D) qRT-PCR analysis of Stfa1 mRNA expression levels in BMMs extracted from the femur and tibia of the three groups. (E) Western blots showing Stfa1 protein expression levels in BMMs extracted from the femur and tibia of the three groups. (F) Densitometric analysis of protein levels relative to β-actin, expressed as a percentage of WT mice BMM levels. (G) qRT-PCR assessing the transfection efficiency of BMMs extracted from the femur and tibia of WT mice, transfected with either si-Stfa1 or oe-Stfa1 48 h later. (H) TRAP staining after osteoclast induction with BMMs from WT, si-Stfa1, or oe-Stfa1 groups. (I) Area of osteoclasts in the three groups above. For (C), values are mean ± S.E.M. of 6 determinations per group. ***: P < 0.001 compared with WT mice; #: P < 0.05 compared with Bmi1^−/−^ mice. For (D) & (F), values are mean ± S.E.M. of 3 determinations per group. *: P < 0.05, **: P < 0.01, ***: P < 0.001 compared with BMMs of WT mice; ##: P < 0.01 compared with BMMs of Bmi1^−/−^ mice. For (G) & (I), values are mean ± S.E.M. of 3 determinations per group. *: P < 0.05, ***: P < 0.001 compared with Control; ###: P < 0.001 compared with si-Stfa1 group.

In order to identify the key molecule by which metformin corrects increased osteoclast differentiation in Bmi1 deficient BMMs, we extracted RNA from mandibular tissue of 6-week-old wild-type, normal diet fed Bmi1^−/−^ and metformin-supplemented Bmi1^−/−^ mice, and performed RNA sequencing and bioinformatics analysis. We found 72 differentially expressed genes (Fig. 6A) that were commonly expressed in the three groups and met the significance criteria (pvalue≤0.05 & |log2(fold change)|≥0). Among these genes that were upregulated in normal dietary Bmi1^−/−^ mice and downregulated in metformin-supplemented Bmi1^−/−^ mice, we identified the osteoclast bone resorption promoting factor Stfa1 (Fig. 6B). We validated the RNA sequencing results by using qRT-PCR to detect Stfa1 mRNA levels in mandibular tissues from the three groups of mice (Fig. 6C). Furthermore, we isolated and cultured BMMs from the three groups of mice and used qRT-PCR and Western Blot to determine that Stfa1 mRNA and protein levels were upregulated in Bmi1^−/−^ BMMs compared to wild-type BMMs but downregulated in metformin-treated Bmi1^−/−^ BMMs (Fig. 6D–F). To verify the role of Stfa1 gene in promoting osteoclast differentiation of BMMs, we isolated BMMs from bilateral femurs and tibias of 6-week-old wild-type mice and treated the cells with siRNA and lentivirus, respectively (Fig. 6G). After osteoclast induction, the results showed that knocking down Stfa1 significantly inhibited osteoclast differentiation, while this ability was significantly enhanced in Stfa1 overexpressing cells (Fig. 6H and I). These results indicate that metformin corrects increased osteoclast differentiation in Bmi1 deficient BMMs by downregulating Stfa1 expression.

### 3.7 P53 transcriptionally upregulates Stfa1 expression in BMMs to promote osteoclast differentiation

**Figure 7:**
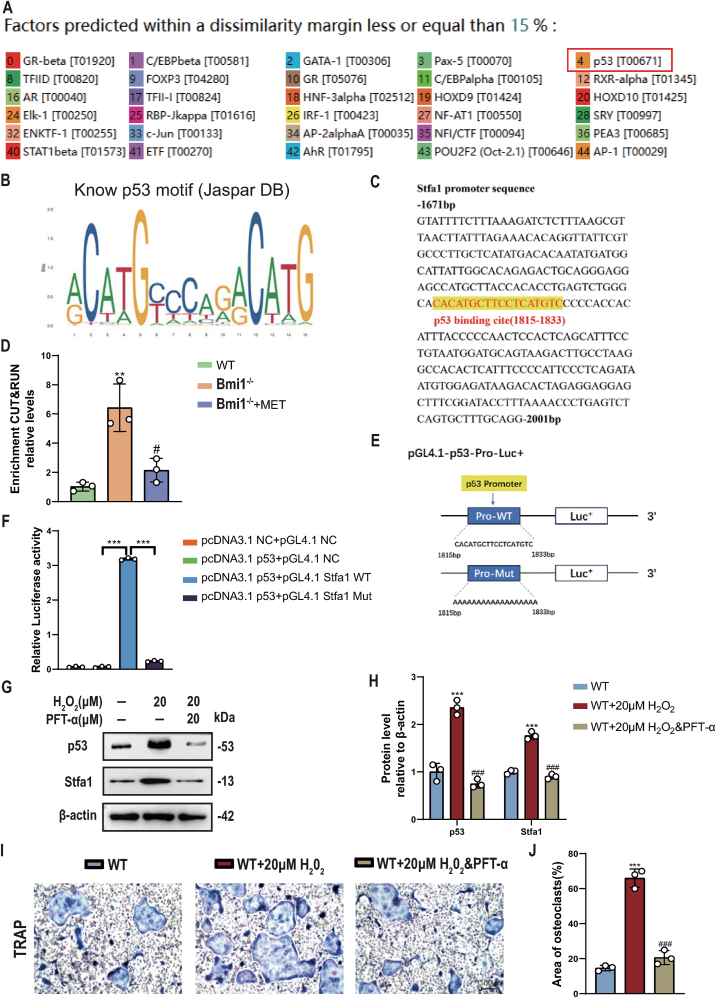
P53 Transcriptionally Upregulates Stfa1 Expression in BMMs to Promote Osteoclast Differentiation (A) PROMO database predictions of upstream transcription factors for Stfa1. (B&C) Prediction of p53 binding sites in the Stfa1 promoter region (yellow region). (D) qRT-PCR detecting the enrichment of Stfa1 in p53 cleavage under targets and release using nuclease. (E) Construction pattern of a luciferase reporter gene plasmid containing the Stfa1 promoter region. (F) Statistical chart of relative luciferase activity. (G) Western blots for p53 and Stfa1 expression levels in BMMs extracted from WT mice and co-cultured with H_2_O_2_ (20 μM) and with or without PFT-α (20 μM). (H) Protein levels relative to β-actin were assessed by densitometric analysis and expressed as a percentage of the levels of BMMs of WT mice. (I) TRAP staining after osteoclast induction in the three groups above. (J) Area of osteoclasts in the three groups above. Values are mean ± S.E.M. of 3 determinations per group. **: P < 0.01, ***: P < 0.001 compared with NC, WT, or pcDNA3.1 p53 + pGL4.1 Stfa1 WT; #: P < 0.05, ###: P < 0.001 compared with si-Bmi1 or WT+20 μM H_2_O_2_.

To investigate whether p53 could directly transcriptionally upregulate Stfa1 expression in BMMs, we predicted the upstream transcription factors of Stfa1 using the PROMO database and found that the transcription factor p53 was among them (Fig. 7A). Bioinformatics analysis revealed 1 potential p53 binding site in the Stfa1 promoter region (Fig. 7B&C). To demonstrate the in vivo physical binding between p53 and the predicted Stfa1 gene promoter binding site, we first designed specific primers targeting the p53 binding site and CUT&RUN-qPCR detection proved that p53 could directly bind to the predicted Stfa1 gene promoter binding site (Fig. 7D). To investigate whether the physical binding of p53 to the Stfa1 gene promoter produces real biological transcriptional regulatory effects, we first constructed the p53 overexpression plasmid (pcDNA3.1-p53), the Stfa1 promoter luciferase reporter plasmid containing the p53 binding site (pGL4.1-Stfa1) and the mutant plasmid (pGL4.1-Stfa1-mutant) (Fig. 7E). After co-transfecting relevant plasmids into 293T cells for 48 h, luciferase activity was detected. The results showed that compared with the control plasmid group, luciferase activity was significantly increased in the group transfected with pcDNA3.1-p53 and pGL4.1-Stfa1 plasmids, while no significant increase was observed in the group transfected with pcDNA3.1-p53 and pGL4.10-Stfa1-mutant plasmids (Fig. 7F). Finally, we treated wild-type BMMs with H_2_O_2_ to model oxidative stress in Bmi1^−/−^ BMMs. This upregulated p53 levels, increased Stfa1 expression, and promoted osteoclast differentiation, whereas inhibiting p53 with 20 μM p53 inhibitor pifithrin-α (PFT-α) prevented these H_2_O_2_-induced effects (Fig. 7G–J). Together, these results indicate p53 directly promotes osteoclast differentiation by transcriptionally upregulating Stfa1 expression in BMMs.

## 4 Discussion

Bmi1 is an epigenetic regulator that promotes stem cell self-renewal and represses cellular senescence pathways via inhibition of the Ink4a/Arf locus [4]. Previous studies found that mice deficient in Bmi1 (Bmi1^−/−^) display multiple progeroid phenotypes including severe osteoporosis, rendering them a model for accelerated skeletal aging [6,7]. Recently, our group showed that Bmi1^−/−^ mice develop severe mandibular osteopenia as early as 5 weeks old, with dramatic thinning of the cortical bone and loss of trabecular alveolar bone [8]. This bone phenotype results from decreased osteoblast bone formation coupled with excessive osteoclast bone resorption in the Bmi1^−/−^ jaw. In the present study, we explored the molecular mechanisms underlying mandibular bone loss in this accelerated aging model and its prevention by the anti-aging drug metformin.

We demonstrate for the first time that loss of Bmi1 in mouse mandible tissue, BM-MSCs and BMMs reduces AMPK phosphorylation and increases mTOR signaling. AMPK senses falling cellular energy levels to limit anabolic processes and stimulate catabolism, while mTOR promotes growth and biosynthesis in nutrient rich conditions [11]. Their balanced regulation maintains homeostasis. Our finding that Bmi1 deficiency shifts this balance towards excess mTOR activity indicates the mandible environment mimics a constant fed state, similar to what is reported to accelerate aging [23].

Metformin treatment was able to restore AMPK-mTOR equilibrium and improve mandibular density in Bmi1^−/−^ mice. While the mandibular effects of metformin have been little studied, a few reports indicate it can mitigate osteoporosis in aged mice [[12], [13], [14]]. Our data now show this protection extends to a genetic model of accelerated jaw bone aging. At the cellular level, we found metformin enhances osteoblast bone formation and attenuates osteoclast differentiation in Bmi1^−/−^ mandible tissue. Enhancing osteoblast function may relate to metformin improving the decreased antioxidant capacity and increased cellular senescence we observed in Bmi1^−/−^ jaw bones, as oxidative stress and aging can impair bone formation [1,24].

Conversely, osteoclasts mediate the elevated bone resorption in elderly patients with osteoporosis [25]. We demonstrate metformin suppresses osteoclast differentiation in Bmi1^−/−^ BMMs by normalizing dysregulated AMPK-mTOR-p53 signaling. Others show p53 stimulates osteoclast activity [26], fitting with its activation downstream of unrestrained mTOR in our model. Our data newly extend this pathway to regulation of osteoclastogenesis and bone loss.

We further identify increased expression of the novel pro-osteoclastogenic factor Stfa1 in Bmi1^−/−^ BMMs, which metformin represses through AMPK-mTOR-p53 signaling. Very little has been reported for Stfa1 thus far. One paper showed Stfa1 overexpression stimulates osteoclastogenesis in BMMs [27], aligning with our gain- and loss-of-function experiments demonstrating Stfa1 drives osteoclast differentiation in primary BMMs. Bioinformatics predicted p53 could transcriptionally activate Stfa1, which we validated using reporter assays and a p53 inhibitor. These data illuminate a new p53-Stfa1 axis promoting bone resorption, although additional study is required to fully characterize Stfa1 in osteoclast biology.

In summary, this study significantly advances understanding of molecular events underlying mandibular osteoporosis in the context of accelerated aging. We demonstrate for the first time that Bmi1 deficiency shifts bone cell AMPK-mTOR balance to a constitutively fed state, accompanied by reduced antioxidant capacity and enhanced cellular senescence. We propose this creates an environment favoring osteoclastogenesis via upregulation of p53 and downstream effector Stfa1, culminating in excessive bone resorption. Metformin corrects AMPK-mTOR dysregulation to suppress excessive catabolism and preserve jaw bone density. These data implicate new targets for maintaining mandible bone health with age.

A limitation is this study utilized only male mice, whereas bone aging has sex-specific characteristics [28]. Follow-up is required to determine if similar protection occurs in females. As well, additional experiments should explore contributions from other cell types like T cells which influence age-related osteoporosis [29]. Clinical studies are also needed to translate these preclinical metformin findings. Small trials reported some bone density improvement in diabetic elderly humans taking metformin [30,31], supporting potential efficacy. However, jaw bones were not analyzed. Rigorously designed trials are warranted specifically measuring mandibular outcomes.

In conclusion, this is the first demonstration that metformin preserves mandible bone integrity in a genetic murine model of accelerated osteoporosis. Our mechanistic data implicate modulation of cellular energy sensors, oxidative stress, and osteoclast differentiation pathways in protective effects of metformin. These findings suggest metformin prophylaxis merits exploration for mitigating aging-associated jaw bone loss to improve oral function and aesthetics in the growing senior population.

## Author contributions

D.M. and A.G. conceived the project. B.L. performed most of the experiments, analyzed, and compiled the data. J.Z., J.Z., X.J. and R.W. helped with experiments. D.M., A.G. and B.L. participated in writing or editing the paper.

## Data availability statement

The datasets generated and analyzed during the current study are available from the corresponding author on reasonable request.

## Declaration of generative AI and AI-assisted technologies in the writing process

During the preparation of this work the authors used ChatGTP 3.5 in order to improve language and readability. After using this tool, the authors reviewed and edited the content as needed and takes full responsibility for the content of the publication.

## Declaration of competing interest

The authors declare no conflicts of interest.

## References

[1] Srivastava M., Deal C. (2002). Osteoporosis in elderly: prevention and treatment. Clin Geriatr Med.

[2] Hildebolt C.F. (1997). Osteoporosis and oral bone loss. Dentomaxillofacial Radiol.

[3] Farr J.N., Kaur J., Doolittle M.L., Khosla S. (2020). Osteocyte cellular senescence. Curr Osteoporos Rep.

[4] Jacobs J.J., Kieboom K., Marino S., DePinho R.A., van Lohuizen M. (1999). The oncogene and Polycomb-group gene bmi-1 regulates cell proliferation and senescence through the ink4a locus. Nature.

[5] Pardal R., Molofsky A.V., He S., Morrison S.J. (2005). Stem cell self-renewal and cancer cell proliferation are regulated by common networks that balance the activation of proto-oncogenes and tumor suppressors. Cold Spring Harbor Symp Quant Biol.

[6] van der Lugt N.M., Domen J., Linders K., van Roon M., Robanus-Maandag E., te Riele H. (1994). Posterior transformation, neurological abnormalities, and severe hematopoietic defects in mice with a targeted deletion of the bmi-1 proto-oncogene. Genes Dev.

[7] Zhang H.W., Ding J., Jin J.L., Guo J., Liu J.N., Karaplis A. (2010). Defects in mesenchymal stem cell self-renewal and cell fate determination lead to an osteopenic phenotype in Bmi-1 null mice. J Bone Miner Res.

[8] Yin Y., Xue X., Wang Q., Chen N., Miao D. (2016). Bmi1 plays an important role in dentin and mandible homeostasis by maintaining redox balance. Am J Transl Res.

[9] Kulkarni A.S., Gubbi S., Barzilai N. (2020). Benefits of metformin in attenuating the hallmarks of aging. Cell Metabol.

[10] Chen S., Gan D., Lin S., Zhong Y., Chen M., Zou X. (2022). Metformin in aging and aging-related diseases: clinical applications and relevant mechanisms. Theranostics.

[11] Dowling R.J., Zakikhani M., Fantus I.G., Pollak M., Sonenberg N. (2007). Metformin inhibits mammalian target of rapamycin-dependent translation initiation in breast cancer cells. Cancer Res.

[12] Yang K., Pei L., Zhou S., Tao L., Zhu Y. (2021). Metformin attenuates H(2)O(2)-induced osteoblast apoptosis by regulating SIRT3 via the PI3K/AKT pathway. Exp Ther Med.

[13] Xie X., Hu L., Mi B., Xue H., Hu Y., Panayi A.C. (2022). Metformin alleviates bone loss in ovariectomized mice through inhibition of autophagy of osteoclast precursors mediated by E2F1. Cell Commun Signal.

[14] Cao F., Yang K., Qiu S., Li J., Jiang W., Tao L. (2023). Metformin reverses oxidative stress-induced mitochondrial dysfunction in pre-osteoblasts via the EGFR/GSK-3beta/calcium pathway. Int J Mol Med.

[15] Ma T., Tian X., Zhang B., Li M., Wang Y., Yang C. (2022). Low-dose metformin targets the lysosomal AMPK pathway through PEN2. Nature.

[16] Chen H., Hu X., Yang R., Wu G., Tan Q., Goltzman D. (2020). SIRT1/FOXO3a axis plays an important role in the prevention of mandibular bone loss induced by 1,25(OH)(2)D deficiency. Int J Biol Sci.

[17] Ji X., Chen H., Liu B., Zhuang H., Bu S. (2023). Chk2 deletion rescues Bmi1 deficiency-induced mandibular osteoporosis by blocking DNA damage response pathway. Am J Transl Res.

[18] Liu H., Guo J., Wang L., Chen N., Karaplis A., Goltzman D. (2009). Distinctive anabolic roles of 1,25-dihydroxyvitamin D(3) and parathyroid hormone in teeth and mandible versus long bones. J Endocrinol.

[19] Wang H., Hu Z., Wu J., Mei Y., Zhang Q., Zhang H. (2019). Sirt1 promotes osteogenic differentiation and increases alveolar bone mass via Bmi1 activation in mice. J Bone Miner Res.

[20] Yang R., Chen J., Zhang J., Qin R., Wang R., Qiu Y. (2020). 1,25-Dihydroxyvitamin D protects against age-related osteoporosis by a novel VDR-Ezh2-p16 signal axis. Aging Cell.

[21] Zhang Y., Chen G., Gu Z., Sun H., Karaplis A., Goltzman D. (2018). DNA damage checkpoint pathway modulates the regulation of skeletal growth and osteoblastic bone formation by parathyroid hormone-related peptide. Int J Biol Sci.

[22] Yang R., Zhang J., Li J., Qin R., Chen J., Wang R. (2022). Inhibition of Nrf2 degradation alleviates age-related osteoporosis induced by 1,25-Dihydroxyvitamin D deficiency. Free Radic Biol Med.

[23] Weichhart T. (2018). mTOR as regulator of lifespan, aging, and cellular senescence: a mini-review. Gerontology.

[24] Tian Y., Ma X., Yang C., Su P., Yin C., Qian A.R. (2017). The impact of oxidative stress on the bone system in response to the space special environment. Int J Mol Sci.

[25] Rachner T.D., Khosla S., Hofbauer L.C. (2011). Osteoporosis: now and the future. Lancet.

[26] Zauli G., Rimondi E., Corallini F., Fadda R., Capitani S., Secchiero P. (2007). MDM2 antagonist Nutlin-3 suppresses the proliferation and differentiation of human pre-osteoclasts through a p53-dependent pathway. J Bone Miner Res.

[27] Wei R., Zhang L., Hu W., Wu J., Zhang W. (2022). CSTA plays a role in osteoclast formation and bone resorption by mediating the DAP12/TREM2 pathway. Biochem Biophys Res Commun.

[28] Kelsey J.L. (1989). Risk factors for osteoporosis and associated fractures. Publ Health Rep.

[29] Saxena Y., Routh S., Mukhopadhaya A. (2021). Immunoporosis: role of innate immune cells in osteoporosis. Front Immunol.

[30] Lu C.H., Chung C.H., Kuo F.C., Chen K.C., Chang C.H., Kuo C.C. (2020). Metformin attenuates osteoporosis in diabetic patients with carcinoma in situ: a nationwide, retrospective, matched-cohort study in taiwan. J Clin Med.

[31] Tseng C.H. (2021). Metformin use is associated with a lower risk of osteoporosis/vertebral fracture in Taiwanese patients with type 2 diabetes mellitus. Eur J Endocrinol.

